# How can restaurants entice patrons to order environmentally sustainable dishes? Testing new approaches based on hedonic psychology and affective forecasting theory

**DOI:** 10.1080/09669582.2023.2274283

**Published:** 2023-10-25

**Authors:** David Fechner, Marion Karl, Bettina Grün, Sara Dolnicar

**Affiliations:** a Business School, Faculty of Business, Economics and Law Colin Clark, The University of Queensland, St Lucia, Australia; b Social Marketing @ Griffith, Business School, Griffith University, Nathan, Australia; c School of Hospitality and Tourism Management, University of Surrey, Guildford, UK; d Institute for Statistics and Mathematics, Vienna University of Economics and Business, Vienna, Austria

**Keywords:** Affective forecasting, hedonic psychology, episodic thinking, menu design, anticipated enjoyment, sustainable consumption

## Abstract

Encouraging restaurant guests to order vegetarian dishes plays a key role in creating a more environmentally sustainable tourism sector. However, for many consumers eating a meat dish is an important aspect of their enjoyment-focused restaurant experience. Identifying new approaches that support restaurants in selling more vegetarian dishes are urgently needed. Drawing from hedonic psychology and affective forecasting theory, this study tests two interventions aimed at directing ordering towards specific vegetarian dishes in a scenario-based survey experiment with 742 consumers. Results show the potential of affective forecasting as a promising psychological mechanism. Displaying an appetising picture of a vegetarian dish on a menu increases stated ordering of the dish because the picture directs consumer attention to the dish and triggers them to imagine eating the dish. Consumers who imagine eating the dish feel stronger anticipated enjoyment of eating it. Adding to the picture an invitation to imagine eating the dish does not further increase the effect. This study explains the psychological mechanism of how a picture of an appetising vegetarian dish changes food choices and provides restaurants with a cost-effective measure to direct ordering towards more environmentally sustainable dishes.

## Introduction

The global food system generated more than 13 billion tons of carbon dioxide equivalent, which represents 26–34% of all anthropogenic greenhouse gas emissions generated between 2010 and 2015 (Crippa et al., 2021; Poore & Nemecek, 2018). Emissions from clearing land, enteric fermentation in ruminants, fertiliser and manure usage account for 81% of food-related emissions (Poore & Nemecek, 2018). With more than 38% of all habitable land used for food production (Poore & Nemecek, 2018), agriculture is responsible for 80% of land clearing and deforestation (Hosonuma et al., 2012), and 80% of biodiversity loss (Campbell et al., 2017). The environmental footprint of the global food system is largely determined by what type of foods are being produced. Animal agriculture accounts for 56% of food-related greenhouse gases, yet it provides only 37% of the total protein and 18% of the total calories produced globally (Poore & Nemecek, 2018). The expansion of animal agriculture threatens the achievement of the Paris Agreement to limit global warming to well below 2 °C compared to pre-industrial levels, even if fossil fuel emissions were immediately halted (Clark et al., 2020). Compared to diets which include 100 g of meat per day, on average, plant-based diets emit 25% less greenhouse gases, require 25% less land and 46% less water (Scarborough et al., 2023). Transitioning to a plant-based diet would reduce food-related greenhouse gas emissions by 49% (Poore & Nemecek, 2018) and provide half of the net emission reductions necessary to achieve the Paris Agreement (Eisen & Brown, 2022).

Encouraging restaurant guests to order vegetarian dishes is important for creating a more environmentally sustainable tourism sector (UNWTO, 2020) and achieving the United Nations Sustainable Development Goal 12: Sustainable Consumption and Production. Restaurants, particularly in developed countries, have great potential to promote plant-based dishes and, in so doing, facilitate the transition to a more environmentally sustainable tourism sector (Higgins-Desbiolles & Wijesinghe, 2019; Hu et al., 2013; Whittlesea & Owen, 2012).

Encouraging restaurant guests to order a vegetarian dish is, however, challenging. Consumers view restaurant visits as special occasions and, for many people, ordering a meat dish is an important element of an indulgent experience because they perceive meat dishes as tastier than vegetarian options (Biermann & Rau, 2020). Even consumers who reduce their meat consumption at home often give themselves permission to treat themselves to a meat dish when dining out (Biermann & Rau, 2020; Kemper & White, 2021). Traditional approaches—such as labels informing consumers of the environmental footprint of different menu items—have therefore shown minimal success in directing ordering towards more sustainable dishes in restaurants and hotels (Babakhani et al., 2020; Cozzio et al., 2022).

An alternative approach—one aligned particularly well with hedonic contexts such as dining out—is to associate desired environmentally sustainable behaviours with enjoyable experiences (Dolnicar, 2020). This approach is firmly grounded within the theoretical framework of hedonic psychology (Kahneman et al., 1999), which postulates that people pursue behaviours that provide enjoyment and increase instant utility. Hedonic psychology explains why consumers prefer ordering unhealthy foods that provide immediate enjoyment over a healthy alternative that offers long-term benefits. Increasing anticipated enjoyment of vegetarian dishes by using indulgent descriptions has shown promising results in increasing consumption of vegetarian dishes in a non-hedonic context: Manipulating the anticipated tastiness of eating vegetarian dishes by using indulgent descriptions increased demand in a university cafeteria and café as well as at a conference lunch held at the university (Turnwald et al., 2017; Turnwald & Crum, 2019), and at workplace cafeterias (Gavrieli et al., 2022).

The aim of this study is to improve our theoretical understanding of how menu designs can be leveraged to entice restaurant patrons to order an environmentally sustainable dish. We conduct a scenario-based experiment to add to the body of knowledge on environmentally sustainable food choices three key contributions. First, we test if an appetising picture of a vegetarian dish increases stated ordering of the dish in a restaurant. The ability of pictures to direct ordering towards vegetarian dishes is important to assess because common menu design approaches proven to increase ordering of meat dishes, such as pictures, may not be effective in directing ordering towards vegetarian dishes. The widely used approach of promoting dishes as “Chef’s Special” or “Dish of the Day”, for example, has shown mixed results using vegetarian dishes (Bacon & Krpan, 2018; dos Santos et al., 2018; Perez-Cueto, 2021; Saulais et al., 2019; Zhou et al., 2019).

Second, building on hedonic psychology and affective forecasting theory (Wilson & Gilbert, 2003) and, in particular, the psychological mechanism of episodic future thinking (Atance & O’Neill, 2001)—mentally pre-experiencing a situation—we provide a theoretical explanation for how the picture directs ordering to the target dish. Specifically, we test if the picture prompts consumers to imagine eating the dish, and whether imagining it is associated with higher anticipated enjoyment. We also test if the picture increases ordering of the target dish by directing attention towards the dish and reducing the cognitive effort required to make a food choice. By testing these different relationships, our study helps uncover the psychological mechanism of food choice and provides a basis for on-site interventions to reduce meat consumption in a hedonic context.

Third, building on the premise of affective forecasting theory that people can better imagine familiar situations because imaginations are built on past memories (Schacter et al., 2017), we assess if consumers’ familiarity with the vegetarian dish influences the extent to which they mentally pre-experience eating the dish.

Although the primary purpose of this study is to improve our theoretical understanding of how meal choices can be influenced in a way that makes them more environmentally sustainable, findings also have immediate practical implications. If appetising pictures of vegetarian meals on restaurant menus influence ordering, food service providers can adopt this measure and thereby actively work towards reducing their food-related carbon emissions. Enticing restaurant patrons to order a vegetarian dish can also have positive indirect environmental impacts because eating a vegetarian dish when dining out may increase consumption of vegetarian dishes at home (Verfuerth et al., 2021). If participants who are more familiar with the target dish imagine eating the dish to a greater extent, restaurant managers can use this insight to decide which dishes to display on the menu. Restaurant managers may also benefit from cost savings by directing ordering towards higher profit margin vegetarian dishes.

## Literature review

### Existing interventions to increase demand for vegetarian menu items

Past attempts to re-direct ordering towards vegetarian foods in hedonic and non-hedonic contexts include cognitive, affective, and behavioural approaches (Bianchi et al., 2018; Cadario & Chandon, 2020). *Cognitive approaches* aim to change what consumers know about the available dishes (Cadario & Chandon, 2020). Examples of cognitive approaches include providing information about the environmental footprint of the dish, the nutritional value and origin of the dish, and whether the food is an organic or non-organic product (Cozzio et al., 2020). *Affective approaches* manipulate emotions to influence food choices without necessary changing people’s knowledge of the available dishes (Cadario & Chandon, 2020) by, for example, leveraging social norm messages (Sparkman et al., 2020) or using indulgent menu descriptions (Turnwald et al., 2017). *Behavioural measures* alter the choice architecture and aim to direct food choices towards specific dishes without necessarily changing what consumers know or feel about the available menu items (Cadario & Chandon, 2020). Increasing the number of available vegetarian dishes (Garnett et al., 2019) or offering vegetarian dishes as default options (Gravert & Kurz, 2021) are examples for behavioural approaches. While the organisation of interventions in different categories provides valuable benefits in understanding which types of measures are effective in changing behaviours, interventions can also simultaneously share characteristics from the cognitive, affective, and behavioural categories (Cadario & Chandon, 2020).

The evidence for the effectiveness of cognitive approaches is mixed. In a university cafeteria, carbon labels reduced sales of high-emissions meat dishes by 2.4 percentage points, leading to a 6% reduction in overall emissions (Brunner et al., 2018). However, carbon emission labelling failed to increase demand for a salad buffet in a hotel (Cozzio et al., 2022). Informing hotel guests that the salads, tomatoes, and cucumbers available at a salad bar are local, organic, and fair trade, significantly increased consumption of these vegetables (Cozzio et al., 2022). Providing information about the calorie, saturated fat, protein, sugar, and salt content of lettuce increased average consumption of lettuce from 7.1 to 16.1 g per hotel guest (Cozzio et al., 2022).

Altering choice architecture is a powerful approach to behavioural change (Thaler & Sunstein, 2008). In non-hedonic contexts, for example, doubling the number of vegetarian items on the menu of a university cafeteria increases the ordering of meatless dishes by eight percentage points (Garnett et al., 2019). Listing vegetarian options at the start of the menu and separating them from meat dishes at counters increases demand for vegetarian dishes by six percentage points (Garnett et al., 2020). Displaying vegetarian options first on the menu and placing them in the most visible position at the counter increases demand by six percentage points (Kurz, 2018).

Altering choice architecture has also proven promising for behavioural change interventions in the hedonic context of vacations (Dolnicar et al., 2019a, 2019b; Knezevic Cvelbar et al., 2021). Offering meat dishes upon request only reduces demand in restaurants by 25 percentage points over three weeks (Gravert & Kurz, 2021). In a survey experiment based on hypothetical scenarios, changing the heading of the vegetarian section on a restaurant menu from “Vegetarian main courses” to “Environmentally friendly main courses for a happy planet” increased stated orders of vegetarian dishes by nine percentage points (Krpan & Houtsma, 2020). Integrating vegetarian options rather than listing them in a separate vegetarian section increased stated ordering by seven percentage points (Bacon & Krpan, 2018).

Affective approaches manipulate emotions to influence food choices (Cadario & Chandon, 2020) by leveraging social norms or suggesting an increase in enjoyment. Using a flyer to inform restaurant patrons that fewer patrons have been ordering meat dishes recently—a signal that not eating meat is becoming socially desirable—increased ordering of vegetarian dishes at a university café by 13 percentage points (Sparkman & Walton, 2017) and by two percentage points at a university burger restaurant (Sparkman et al., 2020). It is, however, unclear if social norms also work in hedonic contexts: Using a social norm message on the menu increased ordering of vegetarian dishes in a fine dining restaurant during lunch, but reduced ordering during dinner (Sparkman et al., 2020) and failed at a hotel salad bar (Cozzio et al., 2020) and in restaurants within retail stores (Çoker et al., 2022). Triggering consumers’ anticipated enjoyment of eating vegetarian dishes aims at changing the traditional perception that vegetarian dishes are less tasty than meat dishes (Biermann & Rau, 2020), a factor known to prevent meat consumption reduction (Rosenfeld & Tomiyama, 2020). At a university cafeteria, indulgent menu descriptions increased demand by 25% compared to a neutral description and 41% compared to a description of the menu items as healthy (Turnwald et al., 2017). Similarly, across five university dining halls, using taste-focussed instead of heath-focussed or neutral labels increased vegetable uptake by 29% and 14%, respectively (Turnwald et al., 2019). At a university conference lunch, using taste- rather than health-focussed labels increased uptake of vegetarian items between 13 and 27 percentage points (Turnwald et al., 2019). Using appealing names instead of neutral descriptions showed mixed results at restaurants (Gavrieli et al., 2022). At self-service, buffet-style cafeterias in workplaces in the United States and Australia appealing names increased the uptake of plant-based dishes but not in Brazil and Singapore (Gavrieli et al., 2022). Overall, increasing the perceived enjoyment of vegetarian dishes presents a promising avenue to change consumer food choices (Cadario & Chandon, 2020).

### Affective forecasting theory and its role in food choice

Affective forecasting is the process of predicting future feelings based on a mental representation of future experiences or situations (Gilbert & Wilson, 2007). This mental representation or mental image of the future needs to be a vivid imagination with detailed sensorial and contextual information (D’Argembeau et al., 2011), which makes it easy for people to predict how they would feel in a specific situation. For instance, the mental representation can include imaginations relating to all five human sensory systems that help pre-experience how the future event will sound, smell, look, or taste like. The second prerequisite feature that mental representations need to fulfil in the affective forecasting process is a first-person perspective which enables people to imagine their own future in an autobiographical way (Bulley & Schacter, 2020). This episodic future thinking is described as “a projection of the self into the future to pre-experience an event” (Atance & O’Neill, 2001, p. 533) and can be understood as the first step of the affective forecasting process. Hassabis and Maguire (2007) provide a detailed discussion on how these mental representations of the future are created using different psychological processes and they emphasise the role of scene construction where a complex and coherent imagination of an event situated in a specific spatial context is created using detailed imagery, predominantly from previous experiences. For instance, to create an imagination of a future restaurant visit, people use the memories from past visits to build a detailed representation that allows them to pre-experience the event. Because creating an episodic imagination requires mental effort, people only use it in situations they perceive as important (Bulley et al., 2019; D’Argembeau & Van der Linden, 2004).

Once the mental representation of the future event is generated, people predict which positive or negative emotions (valence), and which specific emotions such as anger and joy they will feel in the future event along with the intensity and duration of their emotional reaction (Wilson & Gilbert, 2003). While the valence is relatively simple to predict, people often fail to accurately predict the intensity and duration of their future feelings (Morewedge et al., 2005). Most research on affective forecasting concentrates on these biases in predicted feelings and how affective forecasts can be made more accurate (Dunn et al., 2011; Gilbert et al., 1998; Morewedge et al., 2005). Yet, regardless of these biases, affective forecasting still guides decision-making and can be used to influence behaviour (Karl et al., 2021, 2023).

Affective forecasting involves two forms of emotions (Barsics et al., 2016): Anticipatory emotions include feelings such as hope or fear and occur while thinking about desirable or undesirable future events. In contrast, anticipated emotions are predictions of emotions associated with a future event—rather than actually experienced emotions. For example, people may predict how much they will enjoy eating a chocolate cake after their main meal. The anticipated emotional experience of enjoyment is not a fully conscious emotion, but a rapid, automatic affective response (Baumeister et al., 2007).

Food choices are naturally suited for investigating affective forecasting as people need to decide what they choose to eat in the future without being able to try the food (Gilbert et al., 2002). Consumers create mental images of themselves eating the available dishes (i.e. episodic future thinking) to predict how much enjoyment they will derive from each option. These mental images of the food and the future thinking of eating are the prerequisite for affective forecasting and for changing people’s food choices. In a scenario-based grocery shopping task, engaging in an episodic future thinking task, which involved thinking about a health goal, increased healthier food choices (Hollis-Hansen et al., 2019). Imagining eating either a vegetarian or meat dish—which did not differ on their perceived tastiness—was positively associated with the intention to purchase the dish in an online restaurant ordering scenario (Petit et al., 2022). When consumers imagined the process of drinking a green smoothie (low rating of enjoyment) and eating crisps (high rating of enjoyment), only the mental simulation of eating the crisps increased the desire to consume the product (Muñoz-Vilches et al., 2019).

Pictures are particularly effective in eliciting mental simulation of eating food (Jeong & Jang, 2016; Muñoz-Vilches et al., 2019, 2020; Xie et al., 2016) because they allow for fast communication of meaning (Townsend & Kahn, 2014) and help consumers create a mental image of themselves eating the dish, making them potentially more suitable for use in behavioural change interventions than words (Lu & Chi, 2018). However, no studies have experimentally tested whether pictures of vegetarian dishes in a restaurant trigger episodic thinking and direct ordering towards the target dish. Against this background, we established the following two hypotheses:

**H1** An appetising picture of one single vegetarian dish increases stated ordering of that dish (compared to a verbal description only).

**H2** An appetising picture of one vegetarian dish leads to increased episodic thinking about the dish (compared to a verbal description only).

Proactively encouraging consumers to imagine themselves in a future situation can further increase the extent to which the picture triggers episodic thinking and changes their stated behaviour. Adding a call to “Visualize yourself here” to a flyer showing a holiday destination increased consumers’ imagination of being at the shown location (Petrova & Cialdini, 2005). Adding an encouragement to “Imagine yourself…” on an advertisement displaying a picture of a young and healthy woman holding a chicken sandwich increased participants’ mental simulation of themselves being healthy, which led to more positive attitudes towards the dish and higher purchase intention (Jeong & Jang, 2016). Similarly, including the words “Imagine yourself hiking…” on an advertisement for outdoor equipment stimulated participants’ imagination of using the product (Praxmarer, 2011), and a picture of a man running in a park with the words “Imagine yourself running through this park” increased participants’ imagination of running (Escalas, 2004). We, therefore, propose the following hypotheses:

**H3** An appetising picture of one single vegetarian dish with a call to action to imagine eating this dish increases stated ordering of that dish (compared to the picture only).

**H4** An appetising picture of one vegetarian dish with a call to action to imagine eating the dish increases episodic thinking about it (compared to the picture only).

The extent to which people mentally experience future events depends on several factors, such as the ability to recollect previous personal events because images of the future are often based on past experiences (Schacter et al., 2017). Imagining future scenarios occurring in familiar settings—such as at home or in the pub—creates a more vivid subjective mental representation with more sensorial details than thinking of an event taking place in new environments like the jungle or North Pole (Arnold et al., 2011; de Vito et al., 2012; Szpunar & McDermott, 2008), and consequently guides decision-making. Familiarity describes having a good knowledge of something. People can gain familiarity through direct experiences or indirect information sources (Baloglu, 2001), such as reading a review about a restaurant or a certain dish. The assumed relationship between mental simulations, familiarity and decision-making builds on two psychological effects, as outlined in a study on affective forecasting and accommodation choice (Karl et al., 2023): According to the mere-exposure effect (Zajonc, 1968), people prefer things they have been exposed to previously to novel things they have never seen or heard of before. The mere-exposure effect explains the success of repeated advertising or why we tend to like a song the more we hear it. The second effect is the availability heuristic (Tversky & Kahneman, 1973) which assumes that choices are guided by how easily a mental representation of a future event or situation can be created. Generally, these mental simulations are developed quicker and easier if memories of similar events can be recalled—as is the case for familiar situations.

In tourism research, familiarity has predominantly been used as the diametric opposite of novelty to study destination images (e.g. Prentice, 2004; Stylidis et al., 2020), as a tourist characteristic describing the desire or need for known, familiar experiences while travelling (e.g. Karl et al., 2015) or as a construct that can explain tourists’ on-site travel choices (e.g. food choice—Xu & Zeng, 2022). In the context of increasing demand for plant-based foods, studies have suggested that consumers who are more familiar with vegetables and other plant-based foods are more willing to make dietary changes and reduce meat consumption (Graça et al., 2019). Based on these premises, we tested the following hypothesis:

**H5** The more familiar participants are with the target dish, the more the appetising picture of the vegetarian dish—with or without a call to action—increases episodic thinking.

We propose that imagining eating a dish does not only lead to the directly observed increased stated ordering but, rather, increases anticipated enjoyment of eating the dish, which subsequently translates into higher stated ordering.

**H6** Higher episodic thinking is associated with higher anticipated enjoyment.

**H7** Higher anticipated enjoyment is associated with higher stated dish ordering.

### The role of cognitive attention and effort in food choices

The picture on the menu—with or without call to action—may not only trigger consumers’ imagination of eating the dish but may also direct their attention to the dish and reduce their cognitive effort required to make a meal selection. When consumers scan restaurant menus to identify a set of dishes to consider, they do not pay the same amount of attention to all menu items (Wansink & Love, 2014). Meat eaters, for example, tend to skip the vegetarian section of the menu entirely because they do not identify as vegetarians (Krpan & Houtsma, 2020) and instead search the menu for meat dishes (Wansink & Love, 2014). Common strategies to direct attention to high-profit dishes include listing target dishes at the top or the bottom of the menu (Dayan & Bar-Hillel, 2011), presenting dishes in boxes (Feldman et al., 2014), highlighting dishes as “Chef’s Special” (Bacon & Krpan, 2018) and using graphics (Brewer & Sebby, 2021; Guéguen et al., 2012; Hou et al., 2017; Poundstone, 2010). We, therefore, propose the following two hypotheses:

**H8** The appetising picture of the vegetarian dish—with or without call to action—leads to increased attention to the dish (compared to a verbal description only).

**H9** Higher attention to the dish is associated with higher stated dish ordering.

After consumers select a consideration set of dishes, they compare dishes within this set to one another to decide which meal to ultimately order (Wansink & Love, 2014). The decision-making process requires cognitive effort—cognitive resources required to decide—because consumers must process the available information for each dish, reflect on previous experience, and evaluate the benefits of each dish (Cooper-Martin, 1994). Decisions which require a high amount of cognitive effort take more time and are more difficult compared to decisions with a low cognitive effort (Cooper-Martin, 1994). The Elaboration Likelihood Model of persuasion proposes that stimuli can persuade consumers in two different ways which differ in the cognitive effort required to evaluate the stimuli (Petty & Cacioppo, 1986). Processing the stimuli through the central route of persuasion requires a critical evaluation of the available information and a high amount of cognitive effort. The processing of information through the peripheral route of persuasion uses heuristics developed through habitual behaviours and is faster and requires less cognitive effort compared to the central route. The Elaboration Likelihood Model has been used to study how to trigger pro-environmental behaviours, such as picking up litter in a national park (Brown et al., 2010) or ordering a low-emission dish in restaurants (Liu et al., 2022), and how to design and communicate sustainable tourism products (Font et al., 2018). Food choices are often habitual (Vermeir et al., 2020) and consumers use external cues such as images to minimise the cognitive effort required when deciding what dish to order in a restaurant (Wansink et al., 2001). We, therefore, propose that the image—with or without call to action—reduces cognitive effort and test the following hypotheses:

**H10** The appetising picture of a vegetarian dish—with or without call to action—leads to reduced cognitive effort when choosing a dish (compared to a verbal description only).

**H11** Lower cognitive effort when choosing a dish is associated with higher stated dish ordering of the target dish.

Consumers consider several factors when making food choices, including the environmental sustainability, price, and taste of the dish, along with expectations placed on the individual by family and friends and the broader society (Chen & Antonelli, 2020). However, taste is the key factor for most consumers when making food choices in restaurants (Wansink & Love, 2014). The picture directs consumer attention to the target dish and reduces the cognitive effort required to make a food choice, but also increases the anticipated enjoyment. Compared to attention and cognitive effort, we assume that anticipated enjoyment has the strongest association with stated ordering of the target dish. We propose the following hypothesis based on these insights:

**H12** Anticipated enjoyment explains more variation in stated ordering than attention and cognitive effort.

## Method

### Participants, design, procedure

We recruited 850 participants through Prolific Academic, an online participant recruitment platform for academic research. The data obtained from Prolific Academic has been reported to be of higher quality in comparison to alternative platforms because participants are less familiar with scientific research questions and provide more honest responses (Eyal et al., 2022; Peer et al., 2017). We screened for participants who are over 18 years old, have completed at least 85% of surveys to the satisfaction of the researcher, live in Australia, New Zealand, or the United Kingdom and do not follow any specific diet. We selected participants from these three countries because, on average, they eat more than the recommended amount of meat (Ritchie & Roser, 2019) and because English is their main language, minimising language-related survey biases. We removed 108 participants who failed to complete the survey or failed to pass an attention check (requiring them to follow a specific instruction in their response), resulting in a final sample of 742 respondents (48.7% females; Mean_age_ = 38.6 years, Standard Deviation_age_ = 12.9 years).

Participants completed the questionnaire on a desktop computer to ensure they all saw the picture at the same resolution. The questionnaire took approximately four minutes to complete. Each participant received a reimbursement of AU$0.91. The University Human Ethics Committee approved this study (2021/HE000110).

At the beginning of the questionnaire, we asked respondents to imagine going to a restaurant for a 10 am breakfast. We included a picture of a restaurant to assist with the imagination. We then randomly assigned respondents to one of three conditions. The *control* group respondents (*n*** **=** **244) saw a standard menu cover (“Welcome to the Food & Coffee House. We have an all-day breakfast menu”) followed by the full menu of an existing restaurant containing five meat and five vegetarian dishes. Respondents selected a dish to order, then completed the questionnaire (see full questionnaire in Appendix A, Supplementary Material).

Respondents assigned to experimental group #1 (*picture*) (*n*** **=** **248) saw a picture and a basic description of one of three vegetarian dishes (paleo breakfast, acai bowl or smashed avocado) on the cover page of the menu. We used three different dishes as target dishes to avoid results being dependent on one specific vegetarian dish. A professional chef cooked all three meals, and a professional photographer took the pictures to ensure they looked appetising. The description of the paleo breakfast, the acai bowl, and smashed avocado were as follows: “Paleo Breakfast with Grilled Sweet Potato, Spinach, Kale, Avocado, Haloumi, Pine Nuts & Poached Eggs,” “Acai Bowl with Fresh Seasoned Fruit & Homemade Granola.”, “Smashed Avo with Feta, Dukkha, Lemon & Poached Eggs.” After seeing the menu cover page, respondents selected a dish from the menu and completed the questionnaire.

Respondents assigned to experimental group #2 (*imagination*) (*n*** **=** **250) saw the same pictures and menu item descriptions as experimental group *#1 (picture)*. In addition, we explicitly invited them to imagine eating the dish using indulgent language because taste-focussed menu descriptions have been shown to increase ordering of vegetarian dishes in non-hedonic contexts (e.g. Gavrieli et al., 2022) and inviting participants to imagine the future scenario increases episodic thinking (e.g. Escalas, 2004). The descriptions read as follows: “Imagine eating our delicious Paleo Breakfast with Grilled Sweet Potato, Spinach, Kale, Avocado, Haloumi, Pine Nuts & Poached Eggs: An irresistible combination of smells and textures—creamy avocado, juicy egg and some crunchy pine nuts on top. A celebration of flavours!”, “Imagine eating our delicious Acai Bowl with Fresh Seasoned Fruit & Homemade Granola: An irresistible combination of smells and textures—blended acai with a hint of banana, a mix of fresh fruits and some crunchy granola on top. A celebration of flavours!”, “Imagine eating our delicious Smashed Avocado with Feta, Dukkha, Lemon & Poached Eggs: An irresistible combination of smells and textures—creamy avocado, juicy egg and some fresh feta on top. A celebration of flavours!”. As in the *picture* and *control* group, participants then selected a dish from the menu and completed the questionnaire.

### Measures

Binary stated food choice served as dependent variable. Two items measured episodic thinking, following instructions for vivid imaginations in future thinking (Bulley et al., 2019) and phenomenological characteristics measurement of episodic future thinking (D’Argembeau & Van der Linden, 2004). We formulated the questions as follows to fit the meal order context: “How much did you imagine yourself eating the [insert name of target dish] before you made your meal choice?” and “How much did you imagine the taste of the [insert name of target dish] before you made your meal choice?”. We used the average of the two items as our episodic future thinking measure.

We used a pre-existing single item scale to measure anticipated enjoyment (Turnwald et al., 2019): “How much do you think you will enjoy the taste of the [insert name of target dish]?” 27% of respondents indicated that they did not think about the taste of the target dish. We coded these responses with a value of zero in the data set because not thinking about the dish implies not anticipating enjoyment from eating the dish.

Aided and unaided recall of the target dish being on the menu indicated attention. We measured unaided recall by asking respondents to list all dishes that were on the menu, and aided recall by asking them to indicate which of the listed menu items were on the restaurant menu. We randomised the dish order and added two dishes that were not on the original menu. We analysed whether participants recalled the target dish correctly in either the aided or unaided recall task or in both.

We adapted three items from an existing item battery to measure cognitive effort (Cooper-Martin, 1994). To measure time required to make the decision (Cooper-Martin, 1994), we asked participants: “How long did it take you to decide which meal to order?”. We used two items from Cooper-Martin’s scale (1994) to measure difficulty of making the decision and formulated them as follows to fit the meal order context: “How difficult was it for you to decide which meal to order?” and “How hard did you think about which meal to order?”. The average across these items served as measure of cognitive effort. We also measured the time spent to make the food choice in milliseconds using a timer in the online survey to ensure the self-reported answer to how long it took to decide which meal to order correlates with the actual time of making the choice. This objective measure had high congruence with the subjective assessment provided by respondent (Spearman correlation *ρ* = 0.46, 95% confidence interval [0.40, 0.52]). Hence, only the subjective measure of time required to make the food choice was used to obtain a measure of cognitive effort.

Food familiarity, surprise, age, gender, and meat consumption when eating out for breakfast served as control variables. Food familiarity is known to affect people’s responses to attempts of directing ordering towards plant-focussed dishes (Broers et al., 2019) and to affect the mental simulation process (Xie et al., 2016). We measured it as follows (Torrico et al., 2019): “How familiar are you with the [insert name of target dish]?” Being presented with a surprise-eliciting dish can affect food choices (Grace et al., 2022). To control for surprise, we adapted an existing item (Choi & Nisbett, 2000) to fit the meal order context: “How much do you expect to be surprised by the look and taste of the [insert name of target dish]?”. Because meat consumption frequency may affect how consumers respond to menu changes that aim to promote vegetarian food choices (Bacon & Krpan, 2018), participants indicated their meat consumption when dining out for breakfast. We obtained the age in years and gender of each participant from Prolific Academic and used these variables as control variables because women (Rothgerber, 2013) and people aged below 50 years are more likely to follow a vegetarian diet (Allès et al., 2017). Respondents recorded their responses on a slider scale ranging from 0 to 100 for cognitive effort, episodic thinking, anticipated enjoyment, food familiarity, surprise, and meat consumption when eating out to avoid problems associated with ordinal answer formats (Dolnicar, 2013).

We note that it is possible that respondents guessed which dish we wanted them to order (experimenter demand effect). The relatively low proportion of respondents in the experimental groups choosing this option (about 16%) suggests that the experimenter demand effect did not overwhelmingly determine choices. We also note that there is no social desirability to choose a vegetarian dish. Even if some people may have been influenced by such an effect, the design ensures that they are equally distributed across all experimental conditions.

### Data analysis

To assess the hypotheses H1 to H11, we fitted regression models with dependent and independent variables as specified in the hypotheses. The dependent variable being metric or categorical determined whether we estimated a linear or logistic regression model. We included familiarity, surprise, meat consumption when eating out, age, and gender as control variables in the regression model. To test against the null hypothesis that the regression coefficient of the independent variable is zero, we ran a two-sided t-test (for linear regressions) and an asymptotic two-sided *z-*test (for logistic regressions). The null hypothesis was rejected at a significance level of 5%. In instances where the null hypothesis was rejected, we inspected the regression coefficient to assess if the sign is in line with the hypothesised direction of the effect. For H5 we included an interaction term between experimental condition and familiarity and estimated a coefficient capturing the change in slope of familiarity for respondents in one of the experimental groups. Additional logistic regression models were fitted with a vegetarian dish other than the target dish as dependent variable to assess potential spill-over effects of the experiment including the same control variables for either the control and the picture group or the picture and the imagination group.

We visualised results of hypotheses 1–4 and 6–11 using effect plots (Fox, 2003). Effect plots show the estimated mean of the regression along with 95% pointwise confidence for different values of the independent variable. Control variables appear at their mean values (if they are metric) or at their modes (if they are categorical). For logistic regression models, the predicted mean values represent choice probabilities and are plotted on the logit scale.

We investigated hypothesis 12 by fitting a logistic regression model with stated ordering of the dish as the dependent variable; anticipated enjoyment, attention, and cognitive effort as independent variables; and including all control variables. We assessed the contribution of each of the three independent variables by determining the relative change in explained variation in the dependent variable when omitting each of these independent variables in isolation (with the remaining two independent and the control variables included in the model). The squared correlation between observed and fitted values served as measure of explained variation (Mittlböck & Schemper, 1996). We determined 95% confidence intervals for the relative change in explained variation using bias-corrected accelerated bootstrap confidence intervals (Efron & Narasimhan, 2020).

## Results

Results indicate that the appetising picture of one single dish increases stated ordering of that dish (compared to a verbal description only), confirming hypothesis 1 (H1: effect = 1.73, *z*-value = 4.10, *p-*value < 0.001). The appetising picture of the dish also leads to increased episodic thinking about the dish (compared to a verbal description only), as postulated by hypothesis 2 (H2: effect = 13.72, *t-*value = 6.20, *p-*value < 0.001). Adding the call to action to imagine eating the dish using an indulgent description does not have a further effect on stated ordering of that dish compared to the picture alone, as postulated by hypothesis 3 (H3: effect = −0.14, *z*-value = −0.54, *p-*value = 0.59), or on episodic thinking about the dish (compared to the picture only), as postulated by hypothesis 4 (H4: effect = 1.49, *t*-value = 0.67, *p-*value = 0.50). Potential spill-over effects leading to a change in ordering of a vegetarian dish which was not targeted could not be identified (control versus picture group: effect = 0.08, *z*-value = −0.37, *p-*value = 0.71; picture versus imagination group: effect = −0.43, *z*-value = −1.96, *p-*value = 0.05). Higher familiarity did not increase episodic thinking more for the experimental groups, as postulated by hypothesis 5 (H5: effect = −0.12, *t*-value = −1.98, *p-*value = 0.98). The sequence of hypotheses assessing how an increase in episodic thinking is associated with an increase in anticipated enjoyment (H6: effect = 0.87, *t*-value = 29.06, *p-*value < 0.001) and an increase in anticipated enjoyment with an increase in stated ordering of the dish is confirmed (H7: effect = 0.05, z-value = 8.41, *p-*value < 0.001).

Hypotheses 8–11 investigate whether the alternative explanations of appetising pictures directing consumer attention to the target dish and the appetising picture reducing the cognitive load required to choose a menu item explain the increase in stated ordering of the target dish. Showing an appetising picture of the vegetarian dish—with or without an invitation to imagine eating it—is associated with increased attention levels (H8: effect = 1.05, *t*-value = 8.91, *p-*value < 0.001) and the increase in attention level is associated with higher stated ordering of the dish (H9: effect = 0.68, *z*-value = 3.63, *p-*value < 0.001). In contrast, showing a picture of the vegetarian dish—with or without an invitation to imagine eating it—is not associated with the stated cognitive effort required to decide which menu item to choose. Hypothesis 10, therefore, cannot be confirmed (H10: effect = 1.55, *t*-value = 0.77, *p-*value = 0.44). Despite the insignificant association of showing a picture of the dish and the stated cognitive effort required during menu items selection, people who reported lower cognitive effort have ordered the featured vegetarian dish significantly more frequently in the survey, confirming hypothesis 11 (H11: effect = −0.01, *z*-value = −2.25, *p-*value = 0.02). These results suggest that attention is a second pathway by which appetising pictures increase stated ordering, but reduction in cognitive load is not.

**Figure 1.**
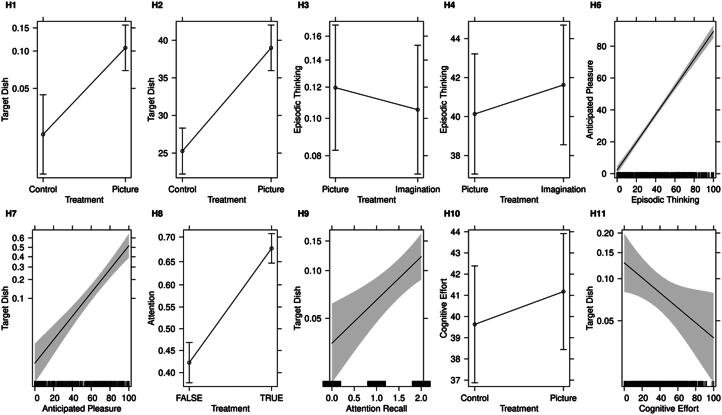
Effect plots for hypothesis H1 to H4 and H6 to H11.

Figure 1 visualises the effects for H1 to H4 and H6 to H11. Each panel relates to one hypothesis and illustrates the association between the dependent and independent variable. As can be seen, the predicted values vary considerably for the hypotheses which were empirically confirmed: The lines connecting the values are steep and the confidence intervals do not overlap, showing the strong influence of the independent on the dependent variables. The negligible impact of the independent variables for hypotheses 3, 4 and 10—which are not supported—is evident from the small difference in the dependent variables and the resulting flat line and the overlapping confidence intervals.

When comparing anticipated enjoyment, attention and cognitive effort with respect to their association with stated ordering of the target dish (H12), anticipated enjoyment emerges as most associated, accounting for more explained variation in stated ordering of the dish than attention and cognitive effort, confirming hypothesis 12. When anticipated enjoyment is omitted from the regression, explained variation drops by 61% (95% confidence interval: [47%, 75%]). When cognitive effort is omitted, explained variation drops by 7% only (95% confidence interval: [2%, 19%]), and when attention is omitted by 4% only (95% confidence interval: [0%, 13%]).

**Figure 2.**
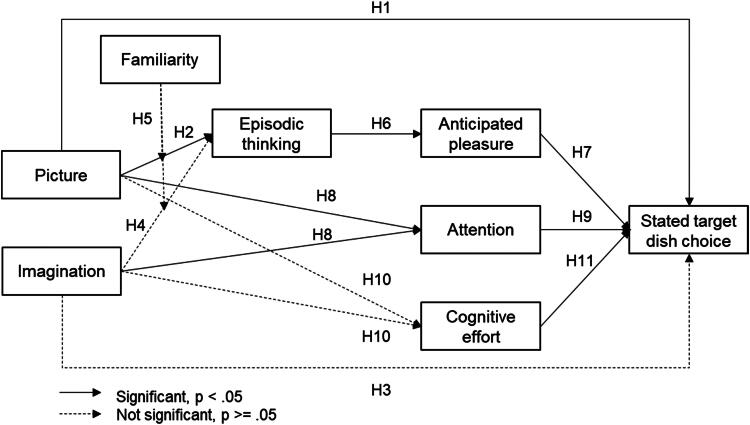
The role of episodic thinking, anticipated enjoyment, and attention triggered by appetising photos in increasing stated target dish choice.

Figure 2 summarises the key findings about the mechanism underlying the effectiveness of appetising pictures of menu items increasing stated ordering of those items. The appetising picture alone—without an additional invitation to imagine eating the target dish—successfully triggers episodic thinking. Episodic thinking is significantly associated with anticipated enjoyment. Anticipated enjoyment is significantly associated with stated choice of the target dish. Adding to the appetising picture also an explicit verbal invitation to imagine eating the dish does not significantly strengthen these causal effects and associations, indicating that the appetising picture is sufficient. Familiarity does not moderate the relationship between the picture of the vegetarian dish—with or without a call to action—and episodic thinking.

## Discussion

Enticing restaurant guests to order vegetarian dishes provides a great opportunity for restaurants to lower their environmental footprint, and in so doing, contributes towards the United Nations Sustainable Development Goal 12. The existing literature suggests that menu design represents a promising avenue for directing ordering in restaurants towards vegetarian dishes (e.g. Demeter et al., 2023; Greene et al., 2023). The aim of this study was to add three contributions to this body of work: (1) determine if an appetising picture of a vegetarian dish increases stated ordering of this dish, (2) provide a theoretical explanation of the psychological mechanism by which the picture directs ordering, and (3) establish if target dish familiarity plays a role in triggering the affective forecasting process.

First, we conclude that an appetising picture of a vegetarian dish on a restaurant menu increases stated ordering of this dish, suggesting that the commonly used menu design approach of using pictures to promote meat dishes (Hou et al., 2017) can also be leveraged to direct meal ordering towards vegetarian dishes. Second, we extend prior findings that identified episodic thinking as an important driver for anticipated enjoyment (Muñoz-Vilches et al., 2020) by showing that imagining eating a vegetarian dish also increases anticipated enjoyment of this dish in a restaurant scenario. Anticipated tastiness is, therefore, not a direct result of the presentation of the dish on the menu. Rather, it is triggered by episodic thinking, as predicted by affective forecasting theory (Wilson & Gilbert, 2003). Importantly: Showing an appetising picture of the vegetarian target dish is sufficient to trigger the mental simulation of eating the dish and does not require a written invitation to imagine eating the dish. This finding is contradictory to prior research, which argues that adding a call to imagine oneself in a specific situation increases mental simulation of a future event (Escalas, 2004; Jeong & Jang, 2016; Petrova & Cialdini, 2005; Praxmarer, 2011). These differences to previous studies may lay in the context of this study and the common daily-life practice of future thinking (D’Argembeau et al., 2011): If people already engage in future thinking every 16 min throughout the day to guide decision-making, an image may already be sufficient to start this process because mental simulation of food consumption is automatically triggered by visual representations of food items (Xie et al., 2016). Previous studies investigated mental stimulation using less habitual behaviours, such as imagining visiting a holiday destination (Petrova & Cialdini, 2005) or hiking in the mountains with certain outdoor equipment (Praxmarer, 2011).

While the picture also increased consumer attention, anticipated enjoyment emerges as contributing substantially more to the explanation of stated dish choice than attention. This means that, while directing attention to vegetarian dishes has a role to play (Bacon & Krpan, 2018), it is the ability of the picture to trigger anticipated enjoyment which appears to ultimately determine consumer choice. Triggering consumers’ imagination of eating a vegetarian dish, therefore, provides a promising avenue to change the common perception that vegetarian dishes are not tasty (Biermann & Rau, 2020). Our study suggests that previous findings from the non-hedonic context (Gavrieli et al., 2022; Turnwald et al., 2017; Turnwald & Crum, 2019) are likely to generalise to the hedonic context: Increasing consumers’ anticipated enjoyment of eating a vegetarian dish may represent a powerful tool for directing consumer food choices.

Third, the results of this study suggest that familiarity with the target dish does not play a role in consumers’ imaginations of eating the dish. Psychological studies on the role of familiarity in episodic future thinking in general have found contrasting results showing that familiar locations have a positive effect on episodic future thinking (Arnold et al., 2011; de Vito et al., 2012). For instance, imagining a common everyday experience in a familiar setting (e.g. going to a lecture at university) elicits stronger episodic future thinking than unfamiliar settings (e.g. being at the North Pole) (de Vito et al., 2012). A potential explanation for the difference in results is that eating in a restaurant represents a highly familiar setting and respondents can easily imagine themselves eating a certain dish. The chosen dishes in our study were common vegetarian meals and results may have been different for novel dishes that respondents have not yet seen in a restaurant, such as cultivated or plant-based meats. An interesting question for future research is whether attempts by restaurants to influence menu choice could backfire in terms of consumers feeling unduly pushed towards choices that they may not see as optimal—an effect recently identified in the contexts of communicating sustainability benefits of products to consumers (Acuti et al., 2022).

Although the purpose of this study was primarily the furthering of theory, findings are also of immediate value to tourism managers, enabling them to reduce their food-related greenhouse gas emissions by redirecting ordering towards vegetarian dishes by including an appetising picture of a vegetarian dish at the front of the menu. Importantly, restaurant managers do not need to add a call to imagine eating the dish to the picture. When deciding which dish to promote, familiarity with the dish is not a critical factor if guests have a basic level of understanding of the dish. In addition to reducing the environmental footprint of their operations (and the tourism sector more broadly), the psychological mechanism uncovered in this study also has the potential to increase the profits of restaurants because many plant-focussed dishes have higher profit margins than meat dishes (Ellis, 2019). To avoid disappointment by patrons who order the target dish, the appetising picture must be representative of dishes served to patrons (Brewer & Sebby, 2021). Creating an attractive and artistic presentation of the ingredients shown in the picture is important because the arrangement of the food on the plate affects how restaurant guests perceive the flavour of the dish before (Michel et al., 2014) and after eating the food (Zellner et al., 2010). Restaurant managers can leverage technology to present the appetising picture of the target dish at various locations. The transition to online ordering in restaurants is a good opportunity to display high-resolution pictures of vegetarian dishes.

This study has a few limitations. First, we measured stated food choices rather than actual behaviour. While survey experiments are commonly used to understand the psychological mechanisms triggered by interventions (because such mechanisms cannot be observed), field experiments are ultimately needed to confirm the effect of the interventions on real behaviour in real-life settings (Viglia & Dolnicar, 2020). Testing the intervention in fast food, casual, and fine dining restaurants would provide valuable insights into the effectiveness of the intervention in different settings which may differ because dining motives vary between restaurant types (Xu & Jeong, 2019).

Second, we instructed participants to imagine visiting a restaurant for breakfast. Because dining time affects the effectiveness of menu changes to entice consumers to order vegetarian dishes (Sparkman et al., 2020), the generalisability of our study findings may be limited to the breakfast setting. Future research could expand the investigation to other mealtimes and test how nudging consumers to order a vegetarian dish at one mealtime affects their food choices later in the day and week.

Third, we collected data from English-speaking consumers. Prior research found different preferences for pictures on menus among English-speaking and Japanese consumers. While Japanese consumers preferred having every dish visually displayed, English-speakers preferred menus showing only pictures of unfamiliar dishes (Verma et al., 1999). Therefore, the present research findings may not be generalisable to cultures where pictures on menus are commonly used. Finally, we tested how displaying one specific vegetarian dish impacts the stated ordering of this dish. Future research could experimentally test how including pictures of multiple vegetarian dishes on the menu simultaneously impacts ordering behaviour. We also used vegetarian dishes which are distinctly different to the available meat dishes. Promoting vegetarian dishes which directly substitute meat dishes, such as a vegetarian burger, lasagne or eggplant parmigiana, may lead to different results.

Fourth, we have not put in place any measures to ensure incentive compatibility of survey responses or account for experimenter demand effects (satisficing) more generally (Krosnick, 1991). One way of doing this in future studies would be to offer respondents, in addition to the small compensation payment for completing the survey, the opportunity to win a free dinner involving the dish they have selected from the menu in the survey.
